# The Ovotransferrin-Derived Peptide IRW Attenuates Lipopolysaccharide-Induced Inflammatory Responses

**DOI:** 10.1155/2019/8676410

**Published:** 2019-01-02

**Authors:** Huanli Jiao, Qing Zhang, Yuanbang Lin, Ying Gao, Peng Zhang

**Affiliations:** General Hospital of Tianjin Medical University, 154 Anshan Road, Heping District, Tianjin, 300052, China

## Abstract

IRW (Ile-Arg-Trp), a bioactive peptide isolated from egg ovotransferrin, has been shown to exert anti-inflammatory effects. In this study, the effects of IRW on inflammatory cytokines and microbiota were explored in human umbilical vein endothelial cells (HUVECs) and a lipopolysaccharide (LPS)-induced rat model of inflammatory peritonitis. Rats were injected intraperitoneally with LPS to establish peritonitis. HUVECs were exposed to IRW for 12 h before introducing LPS. Notably, IRW exerted beneficial effects against LPS-induced peritonitis, specifically, by reducing the serum levels of tumour necrosis factor (TNF)-*α* and interleukin (IL)-6 and myeloperoxidase (MPO) activity (*P*<0.05). A faecal microbiota analysis revealed that IRW significantly increased the Shannon and decreased the Simpson indices (*P*<0.05). Furthermore, IRW treatment significantly inhibited the LPS-induced enhancement of TNF-*α*, IL-8, intercellular cell adhesion molecule-1 (ICAM-1), and vascular cell adhesion molecule-1 (VCAM-1) expression in HUVECs (*P*<0.05). In conclusion, IRW supplementation inhibited the inflammatory mediator synthesis and LPS-induced inflammatory responses and influenced the gut microbiota.

## 1. Introduction

In several diseases, including atherosclerosis, cancer, diabetes mellitus, endotoxic shock, and thrombosis, the pathogenesis is modulated by endothelial cell inflammatory responses [1, 2]. Adhesion molecules and various cytokines, which are key stimulators of the innate immune response and endothelial dysfunction, are typically upregulated during the cellular inflammatory process [3]. Lipopolysaccharide (LPS) is a key membrane component in some Gram-negative bacteria and acts as an important pathogenic stimulus [4] that induces vascular inflammation when introduced into the blood [5]. Specifically, LPS stimulates Toll-like receptor 4 (TLR4) on the surfaces of immune cells to initiate a cascade of downstream signals in human vascular endothelial cells. This cascade leads to the uncontrolled production of cytokines, including tumour necrosis factor (TNF)-*α*, interleukin (IL)-6, and IL-8, as well as adhesion molecules such as intercellular cell adhesion molecule (ICAM) and vascular cell adhesion molecule (VCAM) [6, 7]. The overproduction of these cytokines induces vascular inflammation [8]. Accordingly, inhibiting either the synthesis or release of these inflammatory mediators may limit inflammatory disease.

The unavoidable side effects of synthetic drugs have increased interest in the identification of novel bioactive food components [9, 10]. Many compounds, such as bioactive peptides obtained from foods, are thought to harbour multiple bioactive qualities, including anticarcinogenic, antihypertensive, anti-inflammatory, antimicrobial, and antioxidant activities [11]. IRW (Ile-Arg-Trp), an ovotransferrin peptide isolated from egg white, has been reported to exhibit antioxidant and anti-inflammatory effects both* in vitro* and* in vivo* [12, 13]. However, the anti-inflammatory effects of IRW and the potential mechanisms of action have yet to be determined.

This study explored the anti-inflammatory effects of IRW in an LPS-induced rat model of peritonitis and human umbilical vein endothelial cells (HUVECs). Changes in the faecal microbiota were analysed and the underlying anti-inflammatory mechanisms were considered. 

## 2. Materials and Methods

### 2.1. Reagents and Chemicals

Catalase, dithiothreitol (DTT), Dulbecco's phosphate-buffered saline (DPBS), M199 medium containing phenol red, porcine gelatine, and polyethylene glycol-conjugated superoxide dismutase (PEG-SOD) were purchased from Sigma (St. Louis, MO, USA). IRW was supplied by Ontores Co. Ltd. (Zhejiang, China), and its purity (>99%) was established using high-performance liquid chromatography-tandem mass spectrometry (HPLC-MS/MS). LPS (*Escherichia coli *055:B5) was purchased from Sigma. Enzyme-linked immunosorbent assay (ELISA) kits for TNF-*α*, IL-6, and IL-8 were obtained from the Nanjing Jiancheng Bioengineering Institute (Nanjing, China). Primary antibodies specific for ICAM-1 (ab2213) and VCAM-1 (ab134047) and a horseradish peroxidase (HRP)-conjugated secondary antibody were purchased from Abcam China (Shanghai, China). All remaining reagents and chemicals were of analytical grade.

### 2.2. Animals and Experimental Protocols

This experiment was approved by the Animal Use and Care Committee of the General Hospital of Tianjin Medical University (Tianjin, China). Female adult Wistar–Hannover rats (body weights: 176–200 g) were purchased from the animal experimental centre of Tianjin Medical University. All rats were housed in a humidity- (40–80%) and temperature-controlled room (24°C ± 1°C) with access to food and water* ad libitum*. The rats were acclimatised to this environment for 1 week and maintained on a 12-hour:12-hour light:dark cycle. Thirty-two rats were randomly allotted to 4 groups: control, LPS, IRW, and IRW-LPS. Rats in the control and LPS groups continued to receive basic feed. Rats in the IRW and IRW-LPS groups received an IRW feed supplement (40 mg/kg feed) for 1 week. In the LPS group and IRW-LPS groups, peritonitis was induced by the intraperitoneal injection of LPS (10 mg/kg body weight). Faecal samples were collected from the different rat groups at 3 days after the injection. The rats were then sacrificed; blood was collected via cardiac puncture and divided into whole blood or plasma samples. The white blood cell (WBC) count was measured using a haematology analyser. ELISA kits from Invitrogen (Carlsbad, CA, USA) were used to detect the plasma levels of TNF-*α* (128 tests, catalogue # BMS607-2INST), IL-6 (96 tests, catalogue # KMC0061), and MPO (96 tests, catalogue # EMMPO) according to the manufacturer's instructions.

### 2.3. DNA Amplification and Sequencing

DNA was extracted from 1-g aliquots of faecal samples using the QIAamp DNA Stool Mini Kit (Qiagen, Hilden, Germany) in accordance with the manufacturer's instructions. A UV-vis spectrophotometer (Thermo Scientific, Waltham, MA, USA) was used to determine the final concentration and purity of the DNA. Next, the sample DNA was diluted to 40 ng/*μ*l prior to use as PCR templates. The following primers were used for the PCR amplification of the V3–V4 region of 16S rRNA: 338F (5′-ACTCCTACGGGAGGCAGCAG-3′) and 806R (5′-GGACTACHVGGGTWTCTAAT-3′). The PCR amplification protocol was described in a previous report [14]. The samples were then sequenced by Novogene (Beijing, China) using an Illumina MiSeq platform (Illumina, Inc., San Diego, CA, USA) as described in a previous report [15].

### 2.4. Microbial Diversity Analysis

Trimmomatic was used to trim and quality-filter the raw FASTAQ files, which were subsequently merged using FLASH [15]. The following criteria were applied. (1) Reads were trimmed at any site where the average quality score was <20 over a 50-bp sliding window. (2) Primers were matched precisely to enable 2-nucleotide mismatching, and reads with indistinct bases were eliminated. (3) Sequences that overlapped by >10 bp were merged based upon their overlap sequence. Using UPARSE (version 7.1), operational taxonomic units (OTUs) were clustered using a homology cut-off of 97%. UCHIME was applied to detect and remove chimeric sequences. The taxonomy of each sequence was scrutinised using the RDP Classifier algorithm and evaluated against the Silva (SSU123) database [16]. The ACE, Chao1, and Shannon indices were used to determine the biodiversity of the samples [17, 18].

### 2.5. Cell Cultures

HUVEC cell lines were obtained from ATCC (Manassas, VA, USA). The cells were cultivated in a humidified atmosphere at 37°C with 5% CO_2_/95% air. Cells were treated with various concentrations of IRW 12 hours prior to the LPS challenge (1 *μ*g/ml).

### 2.6. Cell Viability and Cytokine Assays

To assess the effect of IRW on cell viability, a 3-(4,5-dimethylthiazol-2-yl)-2,5-biphenyl tetrazolium bromide (MTT) test was used to identify metabolically active viable cells. Briefly, HUVEC cells were cultured for 24 hours at 37°C and then treated with different concentrations of IRW for 2 hours. Subsequently, 1 *μ*g/ml LPS was added to the cultures, which were incubated for 16 hours. Next, a 5 mg/ml MTT solution was added; the mixtures were incubated for 3 hours at 37°C and then stored overnight in sodium dodecyl sulphate (SDS) buffer (10%) containing 0.01 M HCl. A spectrophotometer (TECAN, Austria) was used to detect the absorbance in each well at 570 nm. These experiments were conducted in triplicate. Additionally, the levels of IL-8 and TNF-*α* in the culture medium were measured using ELISAs according to the user's manuals (Nanjing Jiancheng Bioengineering Institute, Nanjing, Jiangsu, China).

### 2.7. Detection of Adhesion Abilities

VCAM-1 and ICAM-1 levels were evaluated using Western blotting. Cells were collected and lysed in lysis buffer (50 mM Tris-HCl, pH 8.0; 5 mM EDTA; 150 mM NaCl; 1% NP-40; 1 mM PMSF; protease inhibitor cocktail and phosphatase inhibitor cocktail) on ice. The cell lysates were then heated to 95°C, held at this temperature for 15–20 min, and then centrifuged at 10,000 g for 12 min. The supernatants were drawn off, and 20 *μ*g of total protein from each sample was separated using 12% SDS-polyacrylamide and transferred to a polyvinylidene fluoride membrane. The membrane was blocked for 1.5 hours with 5% nonfat milk (Bio-Rad) in Tris-buffered saline with 0.1% Tween-20 (TBST) and then incubated overnight with a primary antibody specific for VCAM-1 or ICAM-1 at 4°C. After 3 washes with TBST (8 min/wash), the membrane was incubated with the HRP-conjugated secondary antibody. GAPDH was also probed to confirm equal protein loading. A cell-cell adhesion assay was performed to detect the influences of various doses of IRW on the adhesion of neutrophils to endothelial cells. The neutrophils were fluorescently labelled as described in a previous report [19]. The following formula was used to calculate the percentage of leukocytes adhering to HUVECs: adherence (%) = (signal of adhesion/total signal) × 100.

### 2.8. Statistical Analysis

All data are shown as the means ± standard errors of the means. The statistical analyses were completed using SPSS software (V22.0) (IBM, New York, NY, USA). The data were analysed using Student's t-test or a 1-way analysis of variance, followed by Duncan's test. A *P* value <0.05 was considered to indicate statistical significance.

## 3. Results

**Figure 1:**
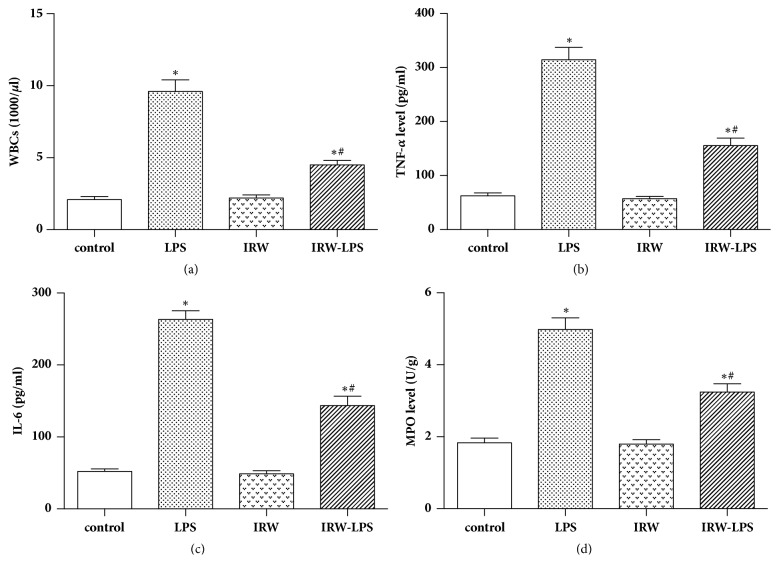
Effects of IRW on (a) the number of WBCs, serum levels of (b) TNF-*α* and (c) IL-6, and (d) MPO activity in a rat model of peritonitis (N=8). *∗ P* <0.05 versus the control group or IRW group. #* P* <0.05 versus the LPS group.

The protective effects of IRW against LPS-induced peritonitis in a rat model were explored by examining the levels of WBCs, TNF-*α*, and IL-6. The results revealed significant reductions in the number of WBCs (*P* <0.05) and the levels of cytokines in LPS-challenged rats treated with IRW (*P* <0.05). Furthermore, IRW inhibited serum MPO activity in rats with peritonitis (*P* <0.05) (Figure 1).

**Figure 2:**
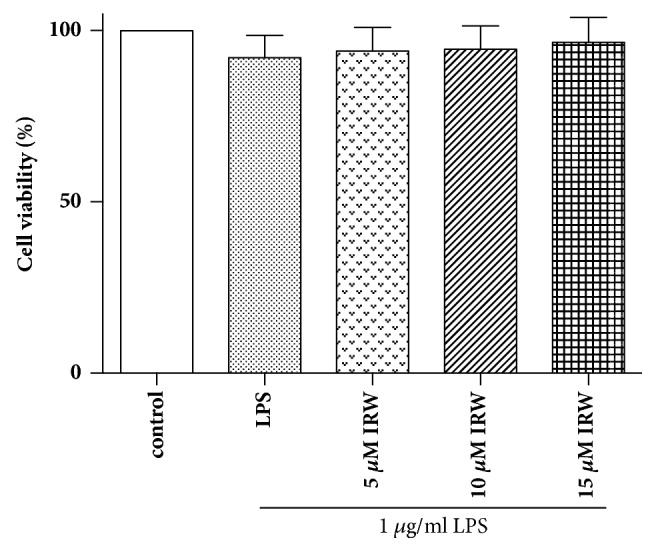
Effects of IRW on HUVEC viability. HUVECs were incubated for 24 hours with LPS and varying concentrations of IRW (5, 10, or 15 *μ*M) (N=3).

An MTT assay was then performed to evaluate the cytotoxicity of IRW.  Figure 2 depicts the cell viabilities at different concentrations of IRW. Consequently, IRW concentrations of 5, 10, and 15 *μ*M were used in further studies.

**Figure 3:**
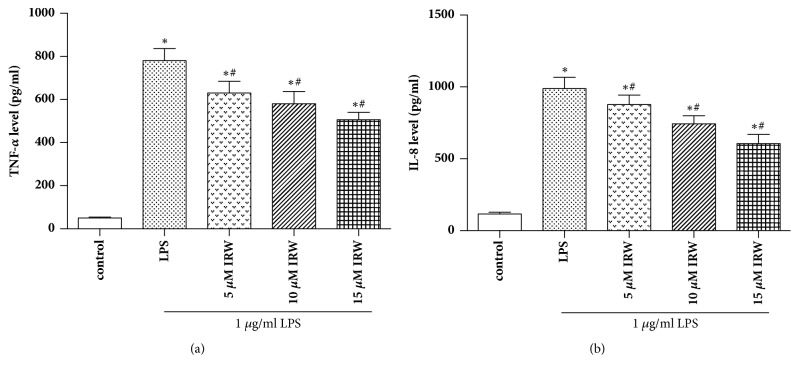
IRW suppressed the synthesis of TNF-*α* and IL-8 in LPS-challenged HUVECs (N=3). *∗ P* <0.05 versus the control group. #* P* <0.05 versus the LPS group.

ELISA was then used to determine the concentrations of TNF-*α* and IL-8 in HUVECs.  Figure 3 shows that the levels of both proinflammatory cytokines were statistically higher in cells incubated with LPS (*P* <0.05). However, these increases were suppressed by IRW in a dose-dependent manner (*P* <0.05).

**Figure 4:**
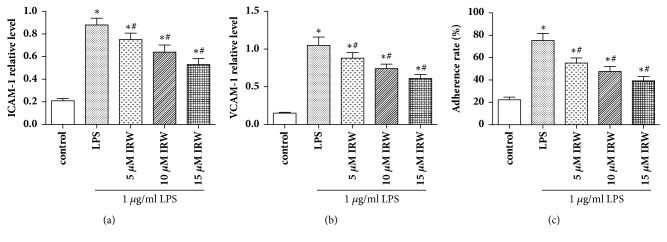
IRW inhibits the expression of (a) VCAM-1 and (b) ICAM-1 and (c) the adhesion of neutrophils to LPS-challenged HUVECs (N=3). *∗ P* <0.05 versus the control group. #* P* <0.05 versus the LPS group.

Western blotting was used to ascertain the impact of IRW on the expression of VCAM-1 and ICAM-1 on LPS-challenged HUVECs. Notably, the levels of both adhesion molecules were increased significantly in LPS-challenged HUVECs (*P* <0.05), while IRW inhibited these increases in a dose-dependent manner (Figures 4(a) and 4(b)). The effect of IRW on neutrophil adhesion rate was also assessed.  Figure 4(c) shows that IRW suppresses the adhesion of neutrophils to HUVECs in a dose-dependent manner.

**Figure 5:**
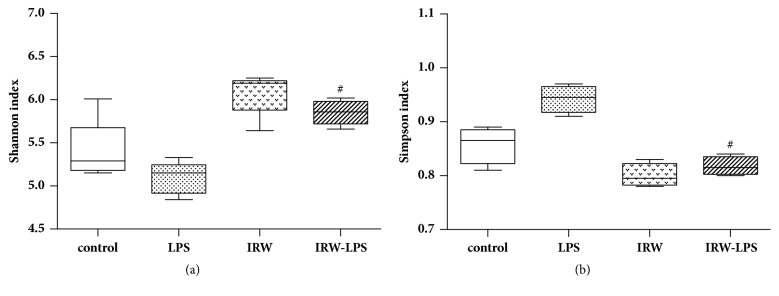
Effect of IRW on the microbial diversity. (a) Shannon diversity index, (b) Simpson index (N=8). #* P* <0.05 versus the LPS group.

**Figure 6:**
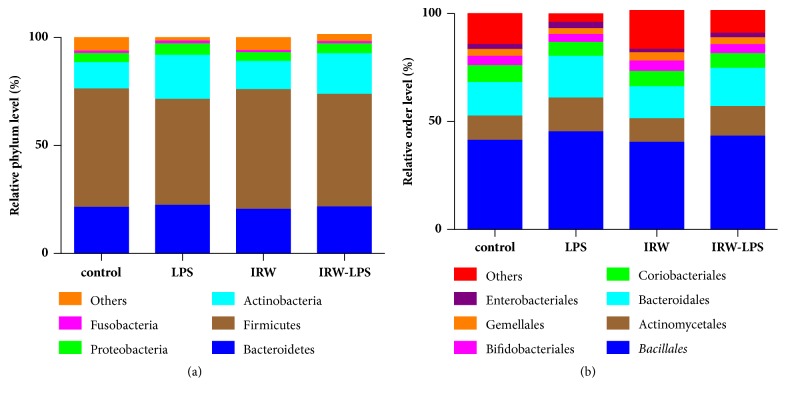
Effect of IRW on faecal microbiota at the (a) phylum and (b) order levels (N=8).

A high-throughput sequencing analysis of gut microbial diversity revealed significant differences in the observed diversities of the Shannon and Simpson indices between the LPS and IRW-LPS groups (Figure 5).  Figure 6 depicts the microbiota composition and relative abundances at the phylum or order level. The faecal samples contained 8 phyla and 19 orders of bacteria. The most abundant phyla were Actinobacteria, Bacteroides, Firmicutes, and Proteobacteria. However, no between-group differences in the relative abundances were detected at the phylum level. The most abundant orders were Actinomycetales, Bacillales, Bacteroidales, Bifidobacteriales, and Coriobacteriales. Similarly, no between-group differences in these relative proportions were detected at the order level.

## 4. Discussion

Food-derived bioactive peptides could potentially be used to treat or even prevent chronic diseases such as diabetes, hypertension, and obesity [20, 21]. This study explored the anti-inflammatory effects of IRW, a tripeptide derived from ovotransferrin, on HUVEC cells and endothelial cells from rats with LPS-induced peritonitis. The study found that IRW exerted its anti-inflammatory and beneficial effects by altering microbial diversity and reducing the synthesis of inflammatory mediators.

The adhesion of monocytes to the endothelium, which is mediated by the interactions of monocytes with adhesion molecules such as VCAM-1 and ICAM-1, represents one of the earliest stages of atherogenesis [22]. These adhesion molecules are key initiators of inflammation and the recruitment of leukocytes to affected sites. Considerable evidence indicates that LPS or TNF-*α* can upregulate the expression of VCAM-1 and ICAM-1 [23]. As we have demonstrated in this study, the increased adhesion of neutrophils to HUVECs was reduced by IRW. Furthermore, the upregulation of VCAM-1 and ICAM-1 in LPS-challenged HUVECs was inhibited by IRW in a dose-dependent manner.

The results of this study reveal that LPS-induced peritonitis could be inhibited by IRW via the suppression of inflammatory cytokine synthesis. Furthermore, this study found that the synthesis of TNF-*α* and IL-8 by LPS-challenged HUVECs was inhibited by IRW in a dose-dependent manner. Research has demonstrated that the overexpression of cytokines is central to the development of inflammation. In particular instances, cytokine overproduction may result in shock, multiorgan failure, or even death [24]. Notably, LPS can upregulate the synthesis of IL-8 and TNF-*α* [25], which, respectively, recruit immune cells to sites of inflammation [26] and induce the synthesis of IL-6, VCAM-1, and ICAM-1 [27, 28]. Therefore, the pharmacological suppression of cytokine overproduction may represent an effective approach to controlling vascular inflammation [29, 30].

As demonstrated by the results of this study, the IRW-LPS group exhibited an increased Shannon index and decreased Simpson index compared with the LPS group. However, no significant differences in the prevalence of organisms were detected at either the phylum or order level. The mechanism by which IRW modifies microbial diversity remains to be established. The majority of studies evaluating the effects of bacteria on inflammatory responses have been constrained by technical challenges that limit the infection model to a single or few aetiologic agents. By contrast, real-world immune systems are exposed to a constant barrage of bacterial species, as well as their metabolites and products [31]. The relationships between immune systems and bacteria have evolved through natural selection over millennia. We therefore propose two potential mechanisms to first address the impact of metabolic modifications on gut microbiota and second explain the potential ability of IRW to improve the barrier function of the intestines. However, more research is needed to determine the mechanisms underlying these effects.

In conclusion, this study has shown that IRW can inhibit inflammatory responses in both HUVECs and a rat model of peritonitis. The observed beneficial effects might be attributable to the ability of IRW to modulate the gut microbiota. These results also support the application of bioactive peptides as functional anti-inflammatory ingredients.

## Data Availability

The data used to support the findings of this study are available from the corresponding author upon request.
